# Can sodium MRI be used as a method for mapping of cartilage stiffness?

**DOI:** 10.1007/s10334-020-00893-x

**Published:** 2020-11-12

**Authors:** Sander Brinkhof, Martijn Froeling, Rob P. A. Janssen, Keita Ito, Dennis W. J. Klomp

**Affiliations:** 1 Department of Radiology, University Medical Center Utrecht, Utrecht, The Netherlands; 2 Department of Orthopaedic Surgery and Trauma, Maxima Medisch Centrum, Eindhoven, The Netherlands; 3 Fontys University of Applied Sciences, Eindhoven, The Netherlands; 4 Orthopaedic Biomechanics, Department of Biomedical Engineering, Eindhoven University of Technology, Eindhoven, The Netherlands; 5 Department of Orthopaedics, University Medical Center Utrecht, Utrecht, The Netherlands

**Keywords:** MRI, Cartilage, Stiffness, Sodium

## Abstract

**Objective:**

Sodium concentration is responsible for (at least part of) the stiffness of articular cartilage due to the osmotic pressure it generates. Therefore, we hypothesized that we could use sodium MRI to approximate the stiffness of cartilage to assess early cartilage degeneration.

**Methods:**

Four human tibial plateaus were retrieved from patients undergoing total knee replacement (TKR), and their cartilage stiffness mapped with indentation testing, after which samples were scanned in a 7 T MRI to determine sodium concentration. The relation of biomechanical parameters to MRI sodium and glycosaminoglycan (GAG) concentration was explored by a linear mixed model.

**Results:**

Weak correlations of GAG concentration with apparent peak modulus (*p* = 0.0057) and apparent equilibrium modulus (*p* = 0.0181) were observed and lack of correlation of GAG concentration versus MRI sodium concentration was observed. MRI sodium concentration was not correlated with apparent peak modulus, though a moderate correlation of MRI sodium concentration with permeability was shown (*p* = 0.0014).

**Discussion and conclusion:**

Although there was correlation between GAG concentration and cartilage stiffness, this was not similar with sodium concentration as measured by MRI. Thus, if the correlation between MRI sodium imaging and GAG concentration could be resolved, this strategy for assessing cartilage functional quality still holds promise.

**Electronic supplementary material:**

The online version of this article (10.1007/s10334-020-00893-x) contains supplementary material, which is available to authorized users.

## Introduction

Focal cartilage defects are often treated with regenerative medicine therapies, because the body cannot heal these defects itself due to the sparsely distributed chondrocytes throughout the articular cartilage and its avascular nature. Patients with focal cartilage defects suffer from pain and functional impairment, which significantly affects their quality of life [1]. Early identification of these focal cartilage defects is important, since it improves the prognosis and outcome of treatment [2, 3]. Arthroscopy has shown to be an excellent method to analyze articular cartilage, especially the discrimination between healthy cartilage and early degenerated cartilage [4]. This early stage cartilage damage is reflected in the softness of the cartilage under indentation during arthroscopy [5]. Softer cartilage damages faster, since it cannot resist loading as well as healthy cartilage. Unfortunately, arthroscopy is still an invasive method to actually measure cartilage quality. Ideally, one would assess the stiffness of cartilage non-invasively, for instance with MRI, because the extent of cartilage damage is most helpful for treatment planning rather than changing treatment strategy intra-operatively.

Cartilage gains its stiffness, among others, due to its ability to retain fluid and swell, both as a result of its osmotic potential [6]. This Donnan osmotic potential arises due to an imbalance of ion concentration between the external sodium concentration (i.e., in the synovial fluid) and the internal tissue sodium concentration. Due to this osmotic potential, and a restriction for swelling and inflow of water by its collagen network, a hydraulic pressure is maintained in the tissue. The imbalance in sodium concentration is a result of the negative fixed charges on the plentiful glycosaminoglycans (GAGs) in cartilage extracellular matrix [7]. These GAGs are bound to a protein backbone forming a proteoglycan which in turn is aggregated to a hyaluronic acid backbone forming a molecule 100 s of millions of daltons in molecular weight. These proteoglycan aggregates in turn are immobilized by being bound to and entangled in the collagen network of the cartilage extracellular matrix and form the ground substance of cartilage with an extremely low permeability to interstitial fluid flow. Thus, when the cartilage is compressed, the resisting stiffnesses, both immediate and after some time when deformation has become constant, i.e., at equilibrium, are due to the concentration of the GAGs and their fixed charge density (FCD). As electroneutrality is always maintained in the tissue, this FCD would also be reflected in the intratissue sodium concentration, making sodium MRI a possible method to measure the stiffness of cartilage in vivo.

Sodium MRI has been able to show accurate measurements of the FCD in vivo in human cartilage [8]. Sodium measurements in the patellae of healthy volunteers showed an average sodium concentration of 254 mM [9]. When using an inversion recovery pulse for fluid suppression, similar sodium concentrations have been shown in healthy cartilage (249 ± 45 mM) [10]. The same range of sodium concentrations was observed in a comparative study in subjects without (220–270 mM) and with osteoarthritis (170–200 mM) [11].

Given the fact that the sodium concentration is responsible for (at least part of) the stiffness of the articular cartilage and sodium MRI is a proven method for assessment of FCD, we hypothesized that we could extend the usability of sodium MRI to approximate the stiffness of cartilage to gain insight into early cartilage damage. We hypothesize that the changes in GAG result in both changes in sodium content (as measured by MRI) and stiffness changes, thus providing a method to assess stiffness with MRI methods. For that purpose, tibial plateaus were tested for their compressive stiffness by means of a stress–relaxation test using an indenter mimicking an arthroscopic probe and consequently scanned ex vivo with sodium MRI using a clinically relevant scan protocol on a 7 T MRI, to assess whether those two parameters (stiffness and sodium concentration) are related to each other.

## Methods

Four tibial plateaus of women (age range 59–70) undergoing total knee replacement (TKR) were retrieved after their surgery and frozen at − 20 °C. The use of these human donor tissues has been determined by the Medical Ethical Committee Maxima Medisch Centrum to not be subject to the guidelines of the Medical Research Involving Human Subjects Acts (WMO), and have been approved by the institutional review board under METC N16.148. The tibial plateaus were thawed to room temperature before the testing procedures began. The testing procedures were carried out as follows: indentation tests were carried out in a tensile tester (Criterion; MTS, Eden Prairie, USA), after which the samples were scanned in a 7 T MRI scanner (Achieva; Philips Healthcare, Best, Netherlands). Samples were transported to the laboratory afterwards to quantify the GAG content by means of a dimethylmethylene blue assay (DMMB).

### Stress–relaxation tests

Stress–relaxation tests were carried out with a 50 N load cell on each individual plateau. The plateaus were submerged in room temperature phosphate-buffered saline (PBS) in a ceramic container. A stainless steel hemi-spherical tip indenter with a diameter of 2 mm was used, since the objective was to mimic the stiffness which the surgeon feels with an arthroscopic probe during surgery. The indentation protocol started with a deformation rate of 50 µm/s until 0.1 N was reached to ensure tissue contact. After tissue contact, the indenter was advanced another 0.3 mm into the cartilage at 0.5 mm/s and then its position was maintained for 240 s to reach equilibrium.

The stiffness was mapped over the whole tibial plateau (on parts which were covered with cartilage, parts with complete denudation were excluded). The indentation measurement locations were at least 5 mm apart to ensure that there was no overlap in measurement areas. Each indentation location was marked with a waterproof marker and photographed to ensure proper location matching in post-processing.

### MR imaging

The tibial plateaus were placed into a custom-made container (outer casing of polyvinyl chloride (PVC), inner sample holder of polyoxymethylene (POM), dimensions 8.5 by 8.5 by 16 cm) which made it possible to image all tibial plateaus at the same time. The plateaus were submerged in Galden PFPE (perfluoropolyether—MR inert fluid) to ensure no background signal was present while imaging the samples.

These samples were placed into a double tuned proton/sodium coil to acquire sodium images which was built in-house [12]. These sodium images were acquired with a 3D FFE with a Cartesian readout; TE = 1.61 ms; TR = 100 ms; flip angle = verified 90° flip angle; FOV, 120 × 120 × 150 mm^3^; voxel size, 3 × 3 × 3 mm^3^; 25 signal averages; total acquisition time of 17 min and 34 s. A non-selective block pulse of 0.1787 ms was used. Three phantoms containing a known sodium concentration (75, 225 and 300 mM) were placed next to the container to enable conversion to sodium concentration in post-processing. The phantoms were 5 mL Eppendorf tubes filled with natrium chloride in water and were acquired in the same scan/field of view as the scans used in this work.

To correct for partial volume effects, morphological imaging was carried out by means of a fat-suppressed gradient echo (GRE) sequence with the following readout parameters: 3D GRE, SENSE factor of 3 (AP), TR/TE/ΔTE/FA = 48 ms/4.5 ms/7.3 ms/16°, ProSet fat suppression, field of view = 120 × 120 × 150 mm^3^, resolution = 0.3 × 0.3 × 0.3 mm^3^ with a total acquisition time of 4 min and 17 s.

### Biochemical analysis

Biochemical analyses were carried out directly after the MRI measurements on each stress–relaxation indentation location, corresponding to the marked locations on the photographs. A biopsy punch with a 3 mm diameter was used to core full-depth cartilage samples at these locations, after which a scalpel was used to remove the cartilage from the subchondral bone. All cores were blotted dry and weighed before papain digestion solution (250 µg/mL papain, Sigma-Aldrich) was added for sample digestion, i.e., incubated overnight at 60 °C. The digested samples were diluted in PBS and stained with the DMMB staining solution [13]. The extinction was measured photospectrometrically at 525 and 595 nm with shark chondroitin-6-sulfate (Sigma-Aldrich) as a standard, after which the total amount of GAGs was measured by dividing the extinction at 525 nm by the extinction at 595 nm. The GAG concentration per wet weight was used in the data analysis.

### Data analysis

Three biomechanical parameters were derived from the stress–relaxation curves: apparent peak force, permeability and apparent equilibrium modulus. The peak force was derived from the stress–relaxation curve as the maximum (i.e., the end of the linear slope transitioning to the exponential decay). The peak force was then used to calculate an apparent peak modulus, by first dividing the peak force with the nominal cross-sectional area of the contact between the indenter and cartilage (being 1.6 mm^2^ at the indentation depth of 0.3 mm) resulting in stress values. These were divided by the nominal strain, calculated as the indentation depth divided by the height of the cartilage as measured by proton MRI at the indentation location, resulting in an apparent peak modulus. The exponential decay of the stress–relaxation curve was fitted with a third-order exponential fit, which yields three signal fractions (compartments) and three time constants. These time constants represent permeability which was calculated using poroelastic theory with $k = \frac{{z^{2} }}{h\tau }$, where permeability *k* is derived from the cartilage height *z* squared divided by the (apparent) equilibrium modulus *h* and time constant *τ*. This apparent equilibrium modulus was derived from the end point force of the exponential decay curve in similar fashion to the peak modulus.

The sodium images were converted to sodium concentration values by using a calibration curve. Three phantoms with known sodium curves were fitted with a linear function, which was consequently used to correct the data from sodium intensity values to sodium concentration values (in mM). These sodium concentration values were corrected for the assumed 70% of water content [9]. To correct for the likely effect of partial volume effects (since the voxels in the sodium imaging protocol are 3 × 3 × 3 mm^3^ and the cartilage probably thinner than 3 mm), the morphological scans are used to calculate the actual thickness of the cartilage per indentation location. The thickness in voxels was measured at each indentation location and consequently multiplied by the voxel thickness of 0.3 mm, resulting in an articular cartilage thickness in millimeters. These thicknesses are used to correct the sodium concentration values, where the assumption is made that the thickness of the cartilage is the same within a voxel and no detectable sodium being present within the voxel outside the cartilage. The sodium concentration in the cartilage is corrected for relaxation time effects by assuming a short T2* component of 0.8 ms, a long T2* component of 14.8 ms and T2* relaxation time in the liquid state phantom of 19.8 ms [14]. We did not correct for minimal non-uniformities in RF coil sensitivity (see supplementary figure for B1 + × B1− map of the RF coil). In most conditions at zero TE, the short component of the sodium T2* accounts for 60% of the signal, whereas the long component accounts for the other 40% of the signal, expressed by the following equation:

$$S = 0.6 {\text{e}}^{{\left( { - \frac{{{\text{TE}}}}{{T2{\text{short}}}}} \right)}} + 0.4 {\text{e}}^{{\left( { - \frac{{{\text{TE}}}}{{T2{\text{long}}}}} \right)}} .$$

Following this equation, the signal contribution of the short T2* component within the cartilage is roughly 8% and the signal contribution of the long T2* component 97%, adding up to a total signal within cartilage of 0.44. Similarly for the liquid state phantom, the total signal adds up to 0.92. To correct for these differences in relaxation time, the sodium concentration values are corrected by a factor of 2.08.

The sodium concentration values were manually matched to the indentation locations of the stress–relaxation tests for the comparative analysis by using high-resolution pictures of the marked indentation locations. Data analysis was carried out in RStudio (version 1.2.1335; RStudio Inc., Boston, MA, USA). The first step of the analysis was the comparison of GAG concentration with the three biomechanical parameters. Thereafter, the comparison was made between GAG concentration and MRI sodium concentration, which are known to be related to one another. Finally, the main hypothesis was tested with the comparison of MRI sodium concentration values versus the biomechanical parameters.

Correlation coefficients (Pearson’s *r*) were calculated for outcome measures relating to each other. Consequently, generalized linear models were fitted to the same parameters with plateau number as covariate. Data were stratified into severely degraded cartilage (≤ 1.5 mm in height) and moderately degraded cartilage (> 1.5 mm in height).

## Results

**Fig. 1:**
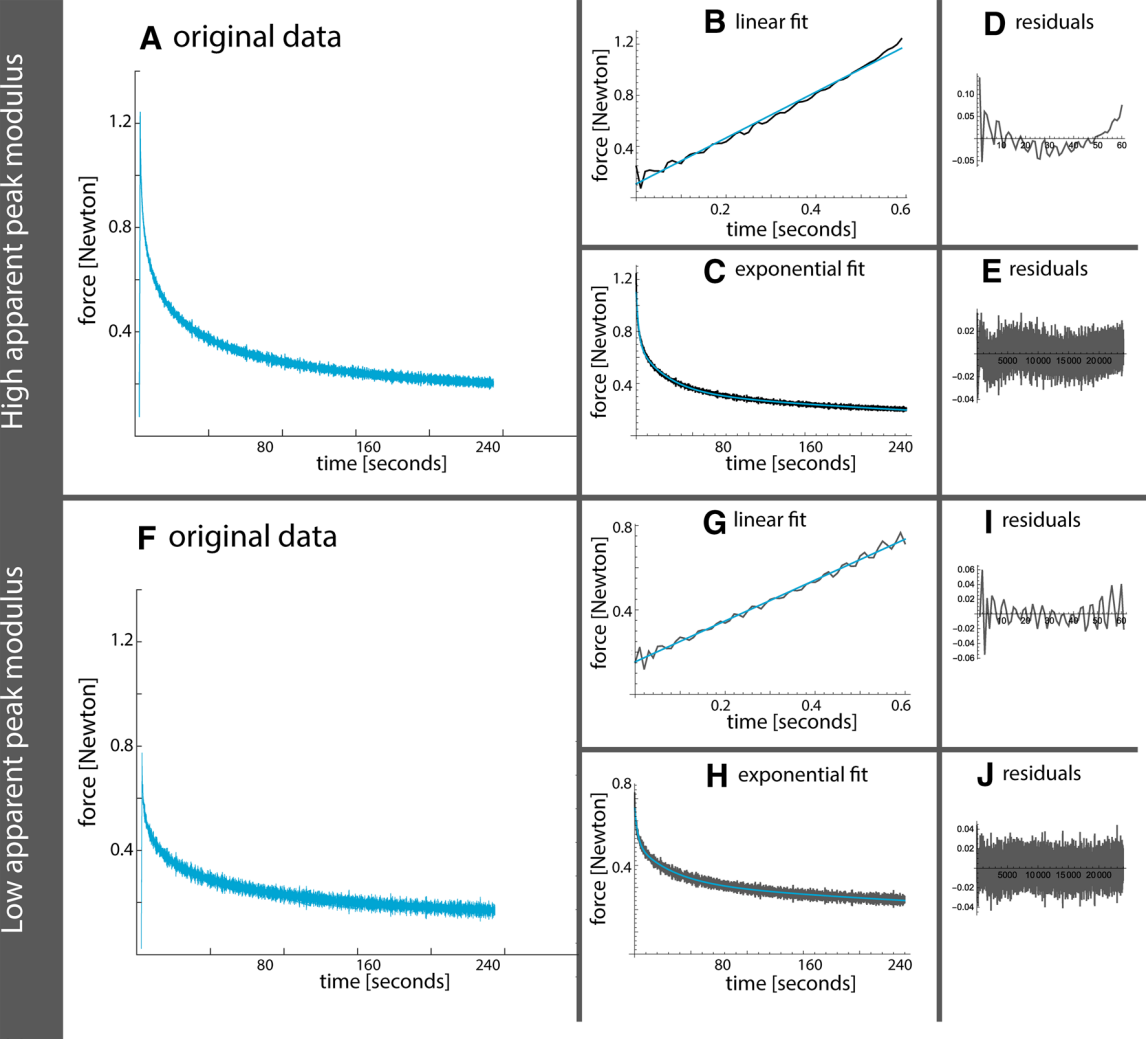
Example of data fits (before conversion to apparent modulus) with a higher apparent peak force (upper part) and lower apparent peak force (lower part). **a**, **f** The original data in blue. **b**, **g** The first linear loading part of the stress–relaxation test with fitted linear regression in blue. Corresponding residuals are shown in **d**, **i**. **c**, **h** The second part of the stress–relaxation test fitted with a third-order exponential decay in blue. Corresponding residuals are shown in **e**, **j**

The four tibial plateaus were subjected to stress–relaxation indentation tests, over 64 locations (range 10–22 per plateau). Figure 1 shows two data examples, one of a sample with a higher apparent peak force and one of a sample with a lower apparent peak force. It can be appreciated that loading and relaxation behaviors of the high force sample do not have a constant pattern in the residuals of the fit.

**Fig. 2:**
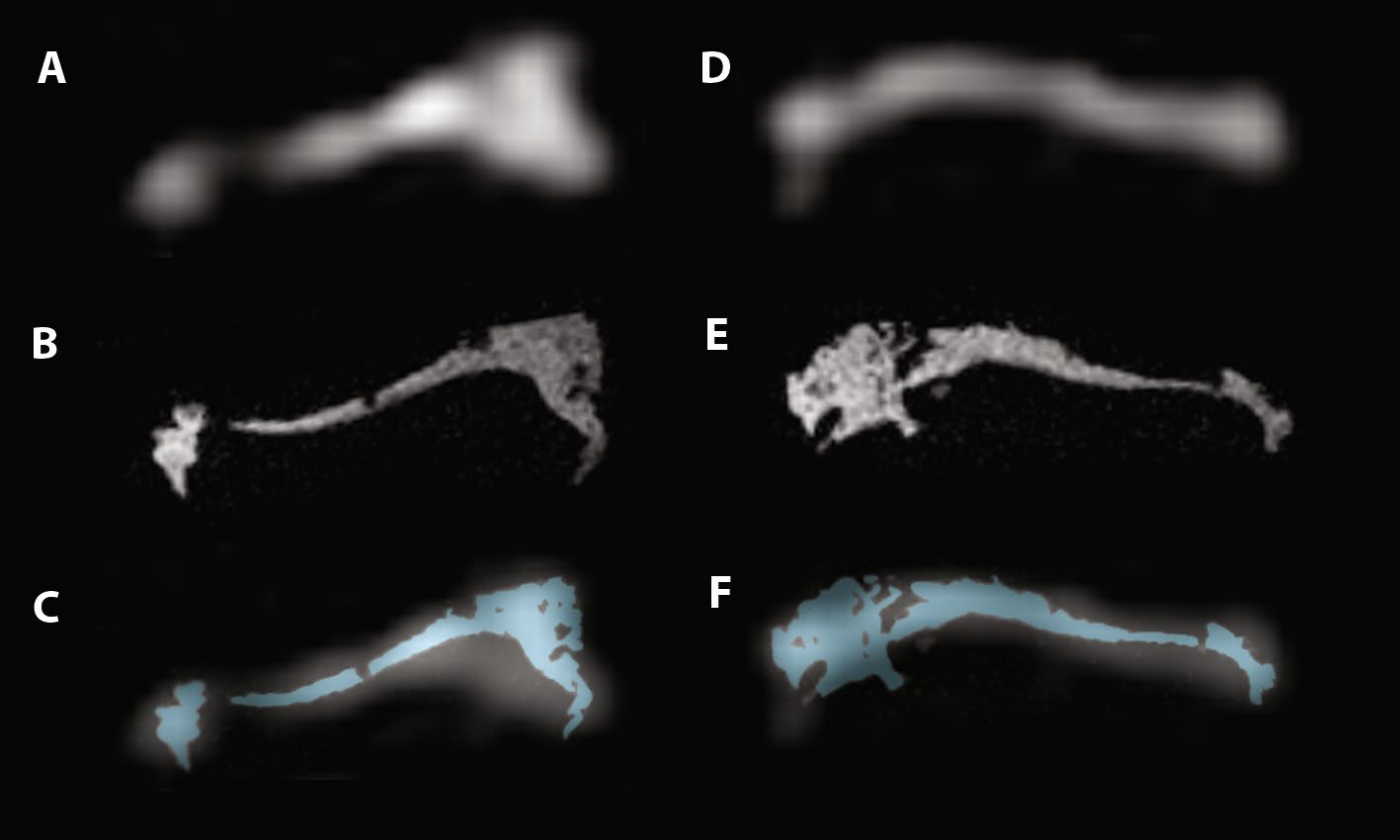
Image examples of two tibial plateaus. Sodium images are shown in **a** and **d**, with their corresponding proton images in **b** and **e**. Pane **c** and **f** The proton images (in blue) overlaid on the sodium scans, which is used for the partial volume correction

Figure 2 shows two MR image examples of tibial plateaus, in which the image quality can be appreciated of both sodium and proton scans. After MRI acquisition, tissue samples were harvested at each indentation location. In total, 50 samples were acquired (range 4–19 per plateau). At 14 indentation locations, no samples were collected due to minimal to no cartilage.

**Fig. 3:**
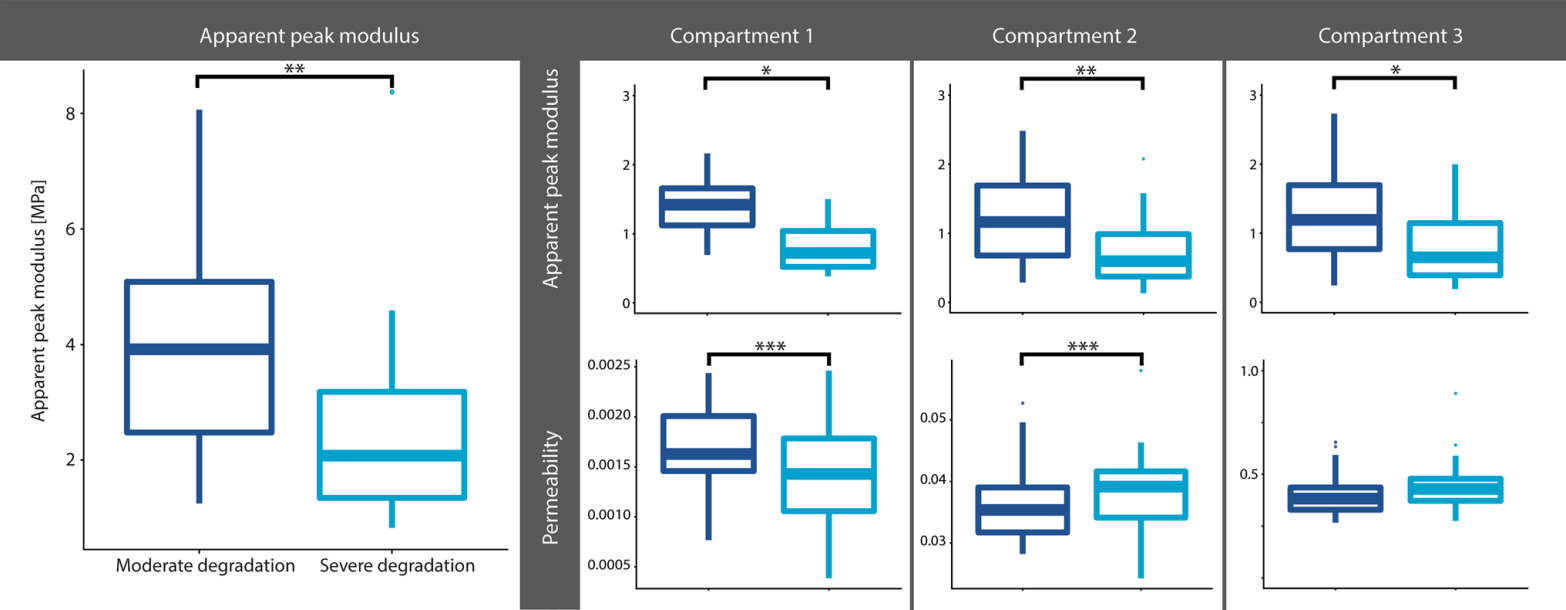
Exploration of difference in fit characteristics as shown in Fig. 1. Note: *p < 0.05, **p < 0.001 and ***p < 0.0001

Figure 3 shows the differences in apparent peak modulus between moderately and severely degraded cartilage. Higher apparent peak moduli are observed in moderately degraded cartilage, whereas lower apparent peak moduli are observed in severe degraded cartilage (*p* < 0.001). These differences between moderate and severe degraded cartilage are also observed in the peak moduli and permeability within the first and second compartment of the exponential fit.

The stress–relaxation tests resulted in a mean apparent peak modulus of 3.36 MPa (range 0.83–8.37 MPa), a mean permeability (compartment 1) of 0.0077 (range 0.0006–0.0198) and a mean apparent equilibrium modulus of 0.81 MPa (range 0.30–2.28 MPa). The sodium 7 T scans resulted in a mean sodium concentration of 224.9 mM over all the plateaus (range 83.8–530.8 mM) and the DMMB assays yielded a mean GAG wet weight content of 3.380 mg/mL (range 0.29–12.96 mg/mL). Table 1 provides an overview of the sodium values obtained in this study.

**Table 1:** overview of sodium values

Plateau number	1	2	3	4
Number of samples	14	18	22	10
Median [ICQ] (mM)	236.7 [140.6–356.9]	179.5 [126.3–234.9]	181.0 [149.2–204.2]	325.8 [260.3–394.0]
Range (mM)	104.8–491.3	96.9–385.0	83.8–314.1	176.1–530.8

**Fig. 4:**
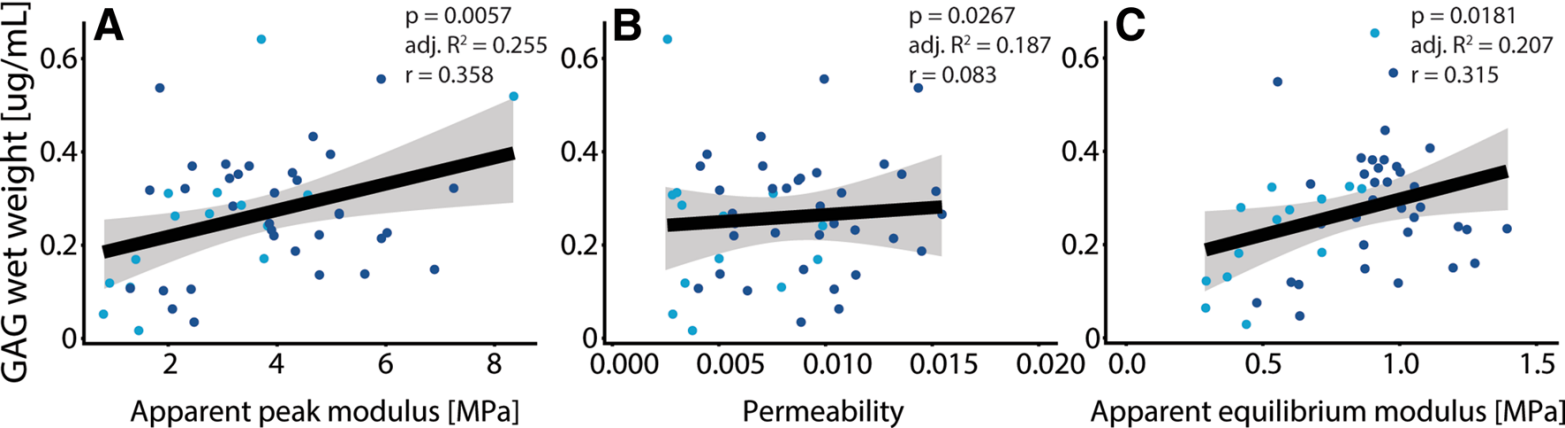
Correlation of GAG concentration versus biomechanical parameters, showing weak correlations of GAG concentration with apparent peak modulus (**a**, *p* = 0.0057) and apparent equilibrium modulus (**c**, *p* = 0.0181). No tangible correlation was observed of GAG concentration versus permeability (**b**)

**Fig. 5:**
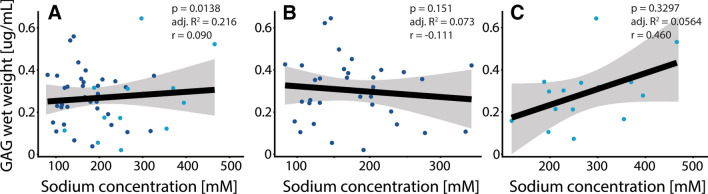
Correlation of GAG concentration versus MRI sodium concentration. **a** Lack of tangible correlation. **b** Moderate degenerated cartilage samples, with no significant correlation. **c** Severe degenerated cartilage samples, with no significant correlation

**Fig. 6:**
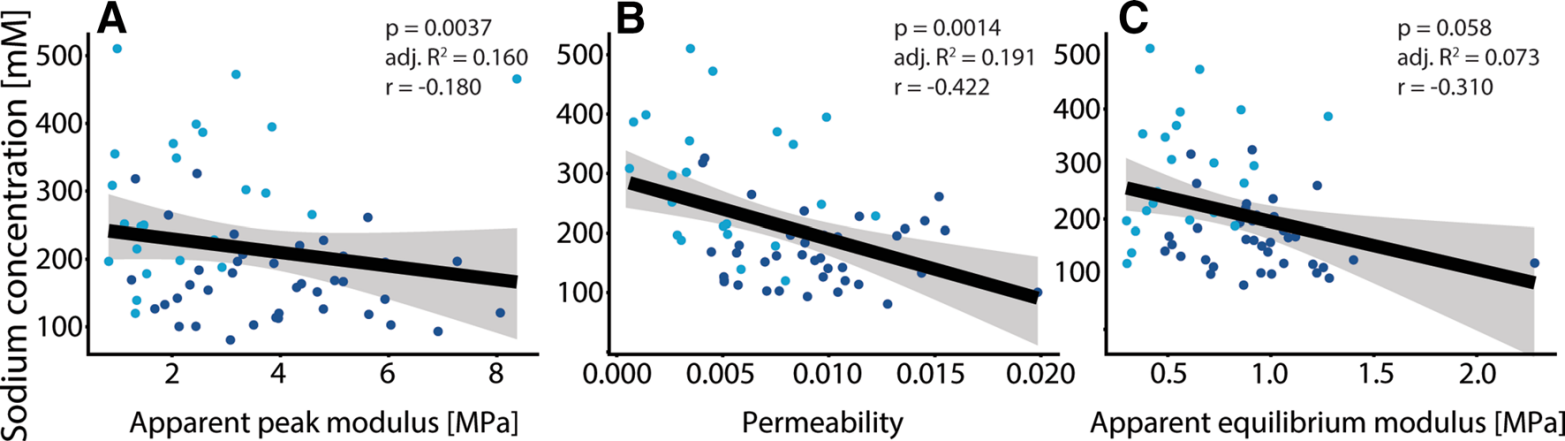
Correlation of MRI sodium concentration versus biomechanical parameters, showing a weak negative correlation of MRI sodium concentration with apparent peak modulus (**a**, *r* = − 0.180, *p* = 0.0037). A moderate negative correlation was observed between MRI sodium concentration and permeability (**b**, *r* = − 0.422, *p* = 0.0014). No significant correlation was observed between sodium concentration and apparent equilibrium modulus

Figures 4, 5, 6 show scatter plots of various outcome measures between one another. The linear model of whole data has been shown with a black line with corresponding confidence intervals in gray. The dark blue dots represent moderate degraded cartilage and light blue dots represent severe degraded cartilage. Figure 4 shows the correlation of GAG concentration with biomechanical parameters, showing weak correlations of GAG concentration with apparent peak modulus (4A) and apparent equilibrium modulus (4C). Figure 5 shows the lack of correlation of GAG concentration versus MRI sodium concentration. Figure 6 shows the correlation of MRI sodium concentration with biomechanical parameters, showing a weak negative correlation of MRI sodium concentration with apparent peak modulus (6A) and a moderate correlation of MRI sodium concentration with permeability (6B).

## Discussion

We hypothesized that we could use sodium MRI to assess the stiffness of the cartilage to gain insight into early cartilage damage. No correlation was observed between apparent peak modulus and MRI sodium concentration, though the permeability was moderately correlated with MRI sodium concentration. GAG concentration was correlated with apparent peak modulus, but the expected correlation between GAG concentration and MRI sodium concentration was lacking.

This study was designed with a clear hypothesis in mind, for which state-of-the-art 7 T MRI was used with a dedicated dual-tuned sodium/proton knee coil, optimized for cartilage imaging. We acquired clinically feasible resolution sodium images (3 mm isotropic) and extremely high-resolution isotropic proton images of 0.3 mm isotropic, fully using the potential of our 7 T MRI. This led to a wide range of sodium concentration values observed on these plateaus, concordant with literature values [11, 15]. The samples underwent thorough assessment on a tensile tester, mapping the entire cartilage surface of the tibial plateaus, leading to a similarly wide range of apparent moduli observed. The apparent equilibrium modulus within this work of 0.82 MPa is in line with the work of Kumar et al., showing an average equilibrium modulus of 0.9 MPa in moderately degraded cartilage (ICRS grade III) [16].

Cartilage stiffness and GAG imaging, more specifically dGEMRIC (delayed gadolinium-enchanced magnetic resonance imaging of cartilage), has been utilized in the past. Other components related to stiffness of cartilage can be imaged, such as the collagen architecture, by using T2(*) relaxation time measurements. These measurements have been shown to contribute to the biomechanical properties of articular cartilage [17–19]. Research of Baldassarri et al. showed differences in stiffness between cartilage specimen which were covered by meniscal tissue and specimen which were not covered [20]. In our work, we do not have pre-TKR images which could confirm whether the menisci were intact. Some of the plateaus had some leftover meniscus, but it is likely that chunks are removed in the surgery. Additionally, the imaging analyses revealed that averaging tissue over a larger volume, as is the case with sodium imaging, possibly masks correlations. Earlier research of the same group found that the correlation decreased when the imaging results were averaged over the full thickness as compared with imaging results averaged over the indentation depth [21]. Given the 3 mm isotropic resolution of the sodium acquisition in this work, it could be that true correlations are masked due to the low imaging resolution. Besides improvements in resolution, there are other changes which may be implemented to further improve the quality of sodium imaging, for which we chose an acquisition method which was available on our system in this work. Inversion recovery has been shown to suppress the signal from free sodium within the synovial fluid, enabling direct quantification of the sodium in the cartilage tissue alone [10]. Other improvements in the sodium acquisition could be to further increase the spatial resolution, which can be done using dedicated sequences such as for instance 3D cones [22], variable echo times gradient echo [23] or density-adapted radial [24] sequences which could be carried out with ultra-short echo time pulses.

The sequence of events in our testing procedures was as follows: first the indentation tests, followed by MR imaging and finally biochemical assays. Ideally, the least invasive test should be the first one to be carried out, in this case MR imaging. Since Galden was used to submerge the plateaus for MR imaging (a thick, oily fluid), we were unsure whether this would influence the indentation tests. Therefore, we chose to do the indentation tests before the MR imaging, with the side note that the stress–relaxation tests could have influence on the integrity of the cartilage. Within this sequence of events, we had to freeze the plateaus twice. This extra freeze–thaw cycle could possibly influence the cartilage properties, although the amount of GAGs should not change, therefore we expect this to be of minimal influence on the results. Additionally, Galden can cause the cartilage to dry out, though we did not assume any interaction between the fluid in the cartilage (and therefore no leakage of sodium) and the Galden.

The stress–relaxation tests were carried out with a spheroid indenter, with a diameter comparable to an arthroscopic probe (diameter of 2 mm). Due to the spheroid nature, the actual indented tissue is of a diameter smaller than 2 mm. The sodium MR images have a voxel size of 3 by 3 mm, which makes the sodium concentration to be measured in the area surrounding the indenter instead of directly underneath it. Since we already measured some of the area surrounding the indentation, we did not opt for averaging the sodium signal in a larger ROI which reduces noise-related errors and lowers the standard deviation. Theoretically, the resolution could be improved by increasing the acquisition time greatly. On the other hand, the SNR could be improved by using different acquisition schemes such as discussed above. The increase in SNR could be invested into a higher spatial resolution. We chose to acquire the sodium MR images with this voxel size because of the acquisition time being clinically feasible, which makes it easier to translate these results to the clinic. For future studies, higher-resolution sodium measurements should be added to these protocols to assess the hypothesis whether the resolution might be the obscuring factor in the relation of cartilage stiffness as measured by indentation and sodium concentration.

A number of post-processing steps are carried out in the conversion of sodium images to sodium concentration values, of which most are manually done. The segmentation, or selection, of voxels on the same location as the indentation tests were carried out manually. Later on in this procedure, a number of conversion steps were taken. Firstly, for the water content correction within this work, we chose a fixed content of 70% of water, which seems reasonable given the osteoarthritic nature of the cartilage. It is possible to measure the water content by using MRI, but given the fact that all cartilage is osteoarthritic we chose a fixed content [25]. Relaxation time effects of the short T2* component are taken into account by assuming a short T2* component relaxation time of 0.8 ms, a long T2* component relaxation of 14.8 ms and T2* relaxation time in the liquid state phantom of 19.8 ms [14].

The final step in conversion to sodium concentration values is the partial volume effects correction. This correction is needed, because cartilage is often thinner than 3 mm (as is the sodium image resolution), meaning that the actual sodium signal comes from a smaller volume. Within each indentation location, the average thickness is measured on the morphological scan with a resolution of 0.3 mm isotropic. This means that a crude measurement (with steps of 0.3 mm, range of 1.2–3.0 mm) of thickness is retrieved, for which the sodium voxels are corrected. The assumption is made that the thickness (which is also used to calculate the strain) is similar throughout the voxel and that the point spread function is neglible, meaning that two voxels do not share the same signal. We acknowledge that this point spread function exists due to FFT reconstruction and some blurring for a small fraction of the signal originates from the very short T2* component, which becomes more dominant if a shorter TE is used. The sodium concentration ranges we find are in concordance with literature, since literature values suggest a range of 170–200 mM for osteoarthritic cartilage [11]. The range of sodium concentrations found in our study is quite large (total range 83.8–530.8 mM over all plateaus, interquartile range 146.0–272.4 mM over all plateaus), but not extraordinary large given ranges found in other sodium studies [15, 26].

The residuals resulting from fitting the stress–relaxation curves seem to be different in samples with a high apparent peak force versus a low apparent peak force. Though we do not expect changing the fitting parameters would lead to different results, the residuals shown a non-constant behavior in the linear fit and the exponential fit. We implemented a third-order exponential fit for the exponential part of the curve, which fits the data nicely with low residuals. Additionally, this third-order exponential fit allows analysis in three compartments which could be related to triphasic models of articular cartilage [6]. Compartments 1 and 2 show the expected trends in permeability, which is expected to become higher with more severe cartilage degradation. This relation is confirmed with the moderate negative correlation of sodium and permeability, as shown in Fig. 6b. These observed differences between both groups in Fig. 2 are the reason behind the stratification of moderate and severe cartilage, for instance in the analysis of sodium versus GAG wet weight. This stratification shows different relations in severely and moderately degraded cartilage, although both not significant relations.

Weak correlation of GAG concentration with apparent peak modulus (*r* = 0.358) and apparent equilibrium modulus (*r* = 0.315) have been observed, which is in line with the expected correlations although weaker. The equilibrium modulus should go down with decreasing GAG concentration, because at equilibrium the load is resisted by the solid modulus and swelling pressure—both directly proportional to GAG concentration. This questions whether a direct GAG MRI measurement should have been applied in this work, which could have been done in theory with gagCEST [27]. However since gagCEST is temperature and pH dependent, this would not have been a good choice in these ex vivo studies because those parameters are hard to keep controlled over various samples. GAG measurements can be carried out in vivo by using T1rho imaging or gagCEST. GagCEST has been shown to be able to discriminate between healthy and degraded cartilage, and even assess the region around cartilage defects [27, 28]. GagCEST is known to be prone to B0 and B1 inhomogeneities, which are even larger in smaller tissue samples as used in this work. These inhomogeneities could be mitigated using advanced post-processing and B1 correction.

The correlation of GAG versus MRI sodium concentration was not observed. Here again, indentation locations (and therefore imaging locations) are manually co-registered with the cartilage samples taken for the assay. Degraded cartilage is often thinner, which makes it difficult to harvest. It could be that parts of the calcified layer of the cartilage are harvested as well, greatly increasing the weight of the cartilage but not increasing the GAG content. However, the stratification based on height did not show better results in thicker (i.e., moderate degenerated) cartilage. Kulkarni et al. did DMMB assays in synovial fluid in various stages of OA, showing that there are basically two categories of end-stage OA patients: one group with very high GAG content (possible active cartilage degeneration process) and the other with significantly low GAG content (completely worn-out cartilage) [29]. This could be an important factor in the lack of correlation in our DMMB analyses versus sodium concentration, since mostly end-stage OA cartilage was included. In the context of this work, the lack of correlation possibly shows that GAG measurements and MRI sodium measurements were potentially measuring different things in the context of biomechanical parameters. The relation of GAG concentration as measured with a DMMB assay and sodium MRI has been established in the past, but on intact animal samples [8, 30] or healthy/early degraded cartilage [31], all showing significant strong correlations. These results question whether sodium MRI could be validated in OA cartilage as included in this study. The heavily degraded cartilage as included in this work leads to a large range of sodium concentrations, as we expected. A similarly large range of values is found within the indentation measurements, further confirming the hypothesis of large variation in cartilage quality. Additionally, a limitation of this work is that we did not include measurement of dry weight of GAG and water content separately, which theoretically could have given more insight into this (lack of) correlation.

The expected trend of a positive correlation of peak modulus versus MRI sodium concentration was not observed. On the contrary, a negative correlation of apparent peak modulus with MRI sodium concentration was observed (though very weak) as shown in Fig. 6a. The MRI sodium measurements did show a negative moderate correlation with permeability (as shown in Fig. 6b), again confirming that sodium MRI potentially measures different phenomena compared to GAG measurements.

In conclusion, sodium MR imaging was not found to be a good method to assess articular cartilage stiffness in this work. GAG MRI measurements such as gagCEST might be a better alternative when interested in peak apparent moduli in vivo, but sodium MRI is a good alternative when interested in permeability of articular cartilage.

## Electronic supplementary material

Below is the link to the electronic supplementary material.

Supplementary Fig. 1 Sensitivity uniformity of transmit and receive of the birdcage RF coil. Pane A shows an acquisition (2D FFE with a cartesian readout; TE = 1.61 ms; TR = 100 ms; flip angle = verified 90 degree flip angle; voxel size, 5 x 5 x 20 mm3) of a sodium phantom (sphere of 12 cm in diameter, filled with 4% sodium chloride). Pane B shows an intensity profile of this image (TIFF 10030 KB)
