# Supplementary figures and images for: Can sodium MRI be used as a method for mapping of cartilage stiffness?
Source: MAGMA. 2020 Nov 12;34(3):327–36. doi: 10.1007/s10334-020-00893-x (PMC8154796; doi:10.1007/s10334-020-00893-x)

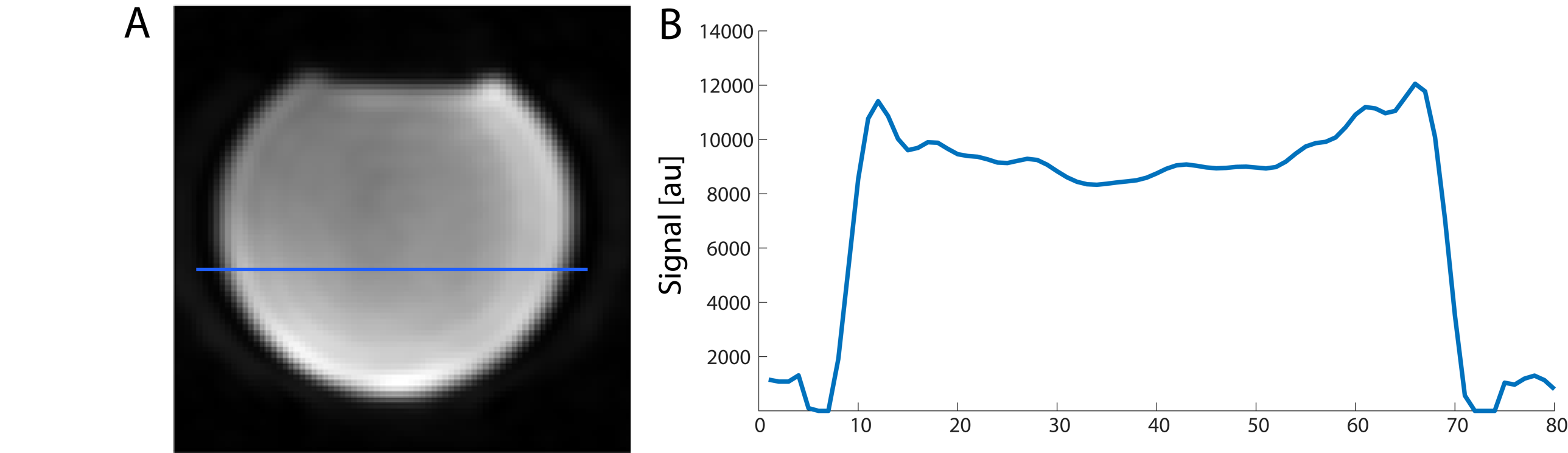

Supplement: Supplementary file 1 — Supplementary Fig. 1 Sensitivity uniformity of transmit and receive of the birdcage RF coil. Pane A shows an acquisition (2D FFE with a cartesian readout; TE = 1.61 ms; TR = 100 ms; flip angle = verified 90 degree flip angle; voxel size, 5 x 5 x 20 mm3) of a sodium phantom (sphere of 12 cm in diameter, filled with 4% sodium chloride). Pane B shows an intensity profile of this image (TIFF 10030 KB) [file 10334_2020_893_MOESM1_ESM.tif]
